# NERO: a biomedical named-entity (recognition) ontology with a large, annotated corpus reveals meaningful associations through text embedding

**DOI:** 10.1038/s41540-021-00200-x

**Published:** 2021-10-20

**Authors:** Kanix Wang, Robert Stevens, Halima Alachram, Yu Li, Larisa Soldatova, Ross King, Sophia Ananiadou, Annika M. Schoene, Maolin Li, Fenia Christopoulou, José Luis Ambite, Joel Matthew, Sahil Garg, Ulf Hermjakob, Daniel Marcu, Emily Sheng, Tim Beißbarth, Edgar Wingender, Aram Galstyan, Xin Gao, Brendan Chambers, Weidi Pan, Bohdan B. Khomtchouk, James A. Evans, Andrey Rzhetsky

**Affiliations:** 1 The Committee on Genetics, Genomics, and Systems Biology, University of Chicago, Chicago, IL 60637 US; 2 The Institute of Genomics and Systems Biology, University of Chicago, Chicago, IL 60637 US; 3 Depatment of Computer Science, University of Manchester, M13 9PL Manchester, UK; 4 Institute of Medical Bioinformatics, University of Göttingen, Goldschmidtstrasse 1, 37077 Göttingen, Germany; 5 Computational Bioscience Research Center, Computer, Electrical and Mathematical Sciences and Engineering Division King Abdullah University of Science and Technology (KAUST) Thuwal, Thuwal, 23955 Saudi Arabia; 6 Goldsmiths, University of London, 8 Lewisham Way, New Cross, London, SE14 6NW UK; 7 Department of Chemical Engineering and Biotechnology, University of Cambridge, Philippa Fawcett Dr, Cambridge, CB3 0AS United Kingdom; 8 Alan Turing Institute, 96 Euston Rd, Somers Town, London, NW1 2DB United Kingdom; 9 Department of Biology and Biological Engineering, Chalmers University of Technology, SE-412 96 Göteborg, Sweden; 10 National Centre for Text Mining, University of Manchester, M1 7DN Manchester, UK; 11 The Information Sciences Institute, University of Southern California, Marina del Rey, CA 90089 US; 12 geneXplain GmbH, Am Exer19b, 38302 Wolfenbüttel, Germany; 13 Knowledge Lab, Department of Sociology, University of Chicago, Chicago, IL 60637 US; 14 Master of Science in Statistics Program, University of Chicago, Chicago, IL 60637 US; 15 Department of Medicine, University of Chicago, Chicago, IL 60637 US; 16 Department of Human Genetics, University of Chicago, Chicago, IL 60637 US

**Keywords:** Software, Diseases

## Abstract

Machine reading (MR) is essential for unlocking valuable knowledge contained in millions of existing biomedical documents. Over the last two decades^1,2^, the most dramatic advances in MR have followed in the wake of critical corpus development^3^. Large, well-annotated corpora have been associated with punctuated advances in MR methodology and automated knowledge extraction systems in the same way that ImageNet^4^ was fundamental for developing machine vision techniques. This study contributes six components to an advanced, named entity analysis tool for biomedicine: (a) a new, Named Entity Recognition Ontology (NERO) developed specifically for describing textual entities in biomedical texts, which accounts for diverse levels of ambiguity, bridging the scientific sublanguages of molecular biology, genetics, biochemistry, and medicine; (b) detailed guidelines for human experts annotating hundreds of named entity classes; (c) pictographs for all named entities, to simplify the burden of annotation for curators; (d) an original, annotated corpus comprising 35,865 sentences, which encapsulate 190,679 named entities and 43,438 events connecting two or more entities; (e) validated, off-the-shelf, named entity recognition (NER) automated extraction, and; (f) embedding models that demonstrate the promise of biomedical associations embedded within this corpus.

## Introduction

Even the relatively specialized subfields of present-day biology and medicine are facing a deluge of accumulating research articles, patents, and white papers. It is increasingly difficult to stay up-to-date with contemporary biomedicine without the use of sophisticated machine reading (MR) tools. MR tool development, in turn, has been limited by the availability of biomedical corpora carefully annotated by experts. This is especially true with respect to information extraction, such as named entity recognition (NER) and relation or event extraction. Although several corpora have been developed for specialized biomedical subdomains, the need for a corpus that can bridge biological, general scientific, environmental, and clinical scientific sublanguages is greater than ever before.

Unfortunately, the annotation of natural science texts is more challenging than in other domains. Biomedical language is replete with ambiguity distinct from that observed in news articles or informal text online. When a word or phrase’s semantic meaning is clearly separated (the east bank of the Danube versus Deutsche Bank), we can implement automated sense disambiguation using machine learning tools. In biomedical texts, however, alternative meanings are not always clearly separated. The problem is not that a phrase can refer to several distinct, real-world entities in different contexts, but that scientists writing articles typically do not separate competing, close meanings. For example, in some biomedical contexts, the words for a named entity may refer to a *gene* or a *protein* with nearly equal probability; for example, “a mutant hemoglobin *α*_2_” can refer to either a gene or a protein. If the author meant *gene-or-protein A*, and we force an annotator to choose either interpretation *gene A* or *protein A*, the resulting annotation is of limited utility because the choice between *gene* and *protein* is random if the meanings are equally likely based on context. Ideally, a specialized ontology of text entities would allow an annotator to choose the proper level of annotation granularity (the words represent a *gene-or-protein*, in this example), minimizing the need for forced, random decisions. To the best of our knowledge, there is no biomedical ontology that meets the requirements for capturing semantic ambiguity. We aimed to fill this gap by developing a specialized, variable-level meaning resolution ontology, a carefully curated corpus, along with corpus annotation tools, and a collection of text embedding analyses to evaluate our annotated corpus.

## Results

**Fig. 1:**
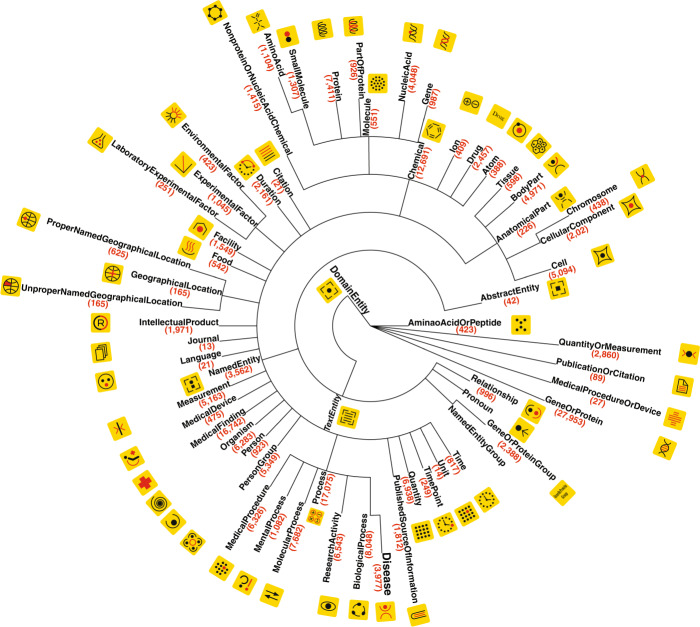
Named Entity Recognition Ontology (NERO). The Ontology is shown here as a multifurcating tree, with taxonomy nodes corresponding to ontology classes. Class name and class mentions count in the corpus are shown in parentheses next to each named entity class. Each taxonomy class is provided with a unique pictogram (black and red shapes on yellow background) intended to simplify expert manual annotation of the corpora. In total, we annotated 35,865 sentences. These sentences encapsulated 190,679 named entities and 43,438 events connecting two or more entities. In addition to the almost two dozen, more sparsely-used branches (such as *ExperimentalFactor* and *GeographicalLocation*) under the *NamedEntity* cluster, there are three heavily-represented branches in our corpus: *AnatomicalPart*, *Chemical*, and *Process*. Slightly more than half (51.6%) of all entities are from these three classes, with 26.6% of all entities originating from *Process* alone. We designed our ontology and its annotations to capture the named entities associated with research activities and facilities; these types of entities can be important for encoding methods used in scientific experiments or patient treatment. The semantic classes *ResearchActivity* and *MedicalProcedures* turn out to be the ninth and the tenth most frequent, respectively. Other top concepts related to the research include *Measurement*, *IntellectualProducts*, *PublishedSourceofInformation*, *Facility*, and *MentalProcess*.

Our new ontology, called NERO, short for Named Entity Recognition Ontology, attempts to minimize unwarranted, arbitrary annotative semantic label assignments for textual entities, see Fig. 1. NERO represents textual entities, and more specifically named entities recognized by text mining tools. NERO is designed as an extension of the IAO (Information Artifact Ontology: http://www.obofoundry.org/ontology/iao.html), one of the widely used OBO ontologies for representing information. The IAO class *InformationContentEntity* is employed as the top-level class in NERO. It has two subclasses: *TextualEntity* defined by IAO and the class *AnnotationInText* to capture information about annotations. The branch *TextualEntity* in IAO includes various parts of text (e.g., Document title, Abstract, and Table), but it does not include entities that are of concern to text mining processes, e.g. *NamedEntity*. NERO enables representations required by text mining and formally defines such classes as *NamedEntity*, *NamedEntityGroup*, *Relationship*, and *Pronoun* as subclasses of the IAO class *TextualEntity*.

NERO defines ambiguous concepts, such as *GeneOrProtein*, which subsumes both *Gene* and *Protein* using the following axiom: *EquivalentTo:* “*Gene*”*or* “*Protein.*” There are no biological entities that are either a gene or a protein, but there are lexical entities that can correspond to either or both of these entities. NERO uses this pattern to express appropriate ambiguity regarding textual entities, preserving uncertainty from the text. In this way, NERO classes represent textual instances and not the actual biological entities to which these instances refer. Often, it is possible to link recognized in the text lexical entities to corresponding biological entities, e.g., through a relationship “is about”.

For example, in some cases the considered previously textual entity “a mutant hemoglobin *α*_2_” can be identified as a protein and linked to the corresponding biological entity defined, e.g., by UniProtKB with URI: U6A3P2. However, as previously discussed sometimes that is not possible. The role of NERO is to provide a logically sound and computationally processable representation of such ambiguous cases.

**Fig. 2:**
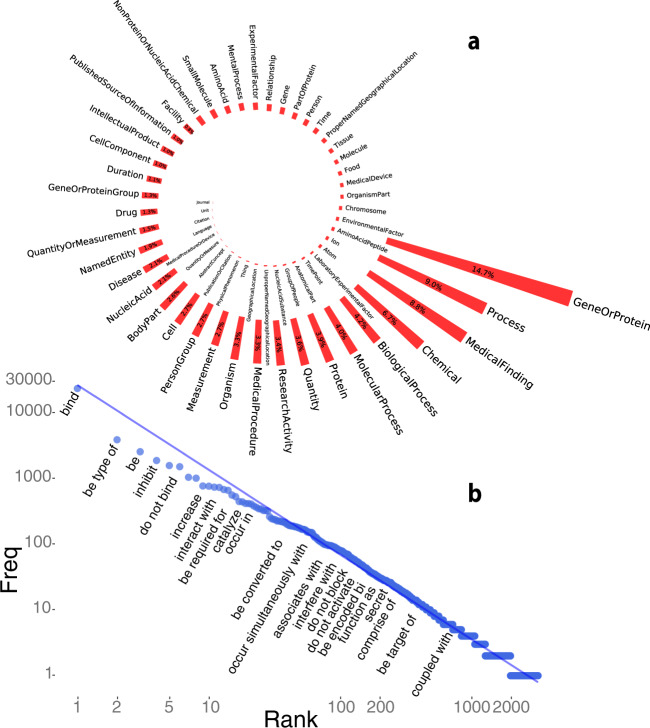
The relative abundance of annotated named entity classes in our corpus. As is typically the case with human languages, semantic classes are represented unevenly in free texts, following a heavy-tail (Zipf’s) distribution. **a** In biomedical corpora, unsurprisingly, named entities associated with *genes* and *proteins* are the most prevalent (15%), followed by *processes* (9%), *medical findings* (8.8%), and *chemicals* (6.7%). At the low-frequency end of the named entity spectrum, we find *journal names*, *units*, *citations*, and *languages*. **b** Events connecting two or more entities are also approximately Zipf-law distributed. Event frequencies are closely tracking corresponding named entity classes. For example, the most frequent event, *bind*, is associated with the most frequently named entity, *GeneOrProtein*. We tried fitting the rank-ordered frequency distribution of annotated named entities with a Discrete Generalized Beta Distribution (DGBD). The result showed a significant deviation from Zipf’s law^33^: The observed distribution’s tail was not heavy enough to match Zipf’s distribution, most likely due to the relatively small number of classes in our ontology^34^. In other words, we expect that frequencies of semantic classes in a very large corpus, annotated with classes from a hypothetical perfect named entity ontology, would follow a Zipfian (discrete Pareto) distribution of named entity classes. Our action annotations have moved beyond interactions between proteins and genes (*e.g*., *bind*, *inhibit*, *phosphorylate*, *encode*), into interactions involving genetic variants and environmental factors (*e.g*., *associated with*, *occur in presence of*, *trigger*, *lack*). Ambiguity levels varied broadly across the named entities captured in our corpus. For example, in the class *AnatomicalPart*, almost all (99.3%) are annotated at the most specific levels, with the majority of entities belonging to *BodyPart*, *CellularComponent*, and *Cell*. In contrast, the general (most vague) concept, *Chemical*, turns out to be the most annotated within its cluster, although more specific subclasses, such as *Protein*, *NucleicAcid*, and *Drug* are also well represented in the corpus. In the *Process* concept cluster, about a third of all concept instances are annotated at a more general *Process* level, and the rest of them are specific concepts, such as *MedicalProcedure*, *MolecularProcess*, *ResearchActivity*, and *BiologicalProcess*. In addition to these major clusters of concepts, several individual concepts are well represented in the corpus. For example, *MedicalFinding* represents 7.3% of all entities. Other well-represented concepts include *Duration*, *IntellectualProduct*, *Measurement*, *Organism*, *PersonGroup*, *PublishedSourceOfInformation*, and *Quantity*. In total, about 70.4% of all entities are annotated at the most specific ontology level. There are five concepts in the NERO ontology that allow the semantic flexibility needed to avoid arbitrary concept assignment. Entities annotated as *AminaoAcidOrPeptide*, *QuantityOrMeasurement*, *PublicationOrCitation*, *MedicalProcedureOrDevice*, and *GeneOrProtein* account for 17.8% of all entities, while less than a quarter (23%) of entities representing either genes or proteins are cleanly annotated with class *Gene* or class *Protein*. The remainder are annotated with class *GeneOrProtein*. In addition to the action *bind*, actions indicating entities’ attributes are the next most frequent. Other biological relationships are also well-represented in this annotation, such as *inhibit*, *activate*, *mediate*, *interact*, *contain*, and *regulate*. The top 30 action categories account for 64.4% of all actions annotated with the top ten action categories accounting for 52.2%. Interestingly, negations of actions were also quite abundant in our annotated corpus. For example, *do not bind* was the sixth most frequent normalized action. Other well-represented negations of actions include *do not affect* and *do not inhibit* (see Supplementary Figs. 1–3).

Striving to make the ontology practically useful, we designed guidelines for annotators making decisions in annotating text entities, available in the Supplementary Data. Furthermore, by recruiting a team of postdoctoral-level and industry experts, we annotated a large biomedical corpus to enable a broad range of natural language processing and biomedical machine learning tasks. Our annotations span 35,865 unique sentences, 8650 of which were annotated by multiple annotators with a remarkably high inter-annotator agreement (see Supplementary Table 1). In our annotated corpus, we aimed to encompass all entity types that might occur in biomedical literature. In addition to named entities, our ontology captures *Events* that represent relationships between biomedical concepts. The frequencies of all diverse entity types in our corpus are shown in Fig. 2A, B shows the frequencies of relations represented in the taxonomy. The most frequent text entity type is *GeneOrProtein*, which accounts for 14.7% of all named entities in the corpus (see Fig. 2A). The second most populous textual entity category is *Process*, with nine percent tagged. *Process* has six sub-concepts and almost half of *Process* instances (49.7%) are annotated as more specific sub-concepts; the *BiologicalProcess* and the *MolecularProcess* are the fifth and seventh most frequent text entity types (see Fig. 2). Entity type frequencies follow a heavy-tail distribution, with the least frequent types being *Journal*, *Unit*, and *Citation* (see Fig. 2). In addition to 190,679 named entities, we annotated 43,438 action terms, events connecting two or more entities. The most annotated action term is *bind*, accounting for 28.4% of all actions, see Supplementary Fig. 1. When we normalize the action terms and combine actions such as *bind*, *binds*, and *binding*, the normalized action *bind* accounts for 31.8% of all actions, as shown in Supplementary Fig. 1. We deployed a package called NERO-nlp for researchers interested in diving deeper into our annotated corpus; the installation guides and scripts are available online at https://pypi.org/project/NERO-nlp and https://github.com/Bohdan-Khomtchouk/NERO-nlp respectively.

Below, we present two practical applications of our ontology and text annotations: (1) Machine learning experiments, which automatically identify named entities, and; (2) Word embedding experiments, which leverage the automated discovery of semantic relationships among real-world concepts referenced by a text’s named entities.

### Machine learning experiments

Using NERsuite^5^, we conducted a tenfold cross-validation, dividing the corpus into training and test subsets. The classification results are presented in Supplemental Tables 1 and 2. The overall automated NER performance is moderate, with 54.9% precision, 37.3% recall, and a 43.4% *F*_1_ score. The best performance class, *GeneOrProtein*, had baseline results of 67.0% precision, 65.3% recall, and a 66.2% *F*_1_ score. In addition to the default baseline implementation of NERsuite, we added additional features in the training process to improve its performance^6^. These are dictionary features derived from lookups in technical term dictionaries. The classifier with dictionary features manifests 54.7% precision, 37.9% recall, and a 43.8% *F*_1_ score. We observed a scant 0.35% increase in *F*_1_ score from adding dictionary features. We then implemented an ensemble method called stacking, where we trained a higher-level model to learn how to best combine contributions from each base model. The base model, in this case, is the baseline model from NERsuite. Stacking yielded a 0.27% increase in *F*_1_ score compared to baseline results. While ensemble methods are commonly used to boost model accuracy by combining the predictions of multiple machine learning models, choices of second-level and base models can influence the amount of improvement in model accuracy. The overall performance statistics are shown in Supplementary Table 2. As our corpus is made public with this study’s publication, we hope that other researchers will use this training data to achieve core MR task performance that surpasses our initial experiments.

To examine how NERsuite performs in comparison to other popular open-source NER tools, we trained a custom NER model on our annotated corpus using spaCy^7^. We evaluated the trained model on the test subset, which consists of a random 10% sample from the corpus. Overall automated NER performance is low, with 30.9% precision, 8.6% recall, and a 13.4% *F*_1_ score. The best performance class, *GeneOrProtein*, had results of 45.1% precision, 36.4% recall, and a 40.3% *F*_1_ score. These statistics indicate a much poorer performance of spaCy compared to that of NERsuite.

To help explain the huge difference in performance between NERsuite and spaCy, we considered the set of input features used by each tool for insight. NERsuite’s baseline implementation uses an extra set of input features including the lemma, POS-feature, and chunk-feature, whereas our custom spaCy NER model only relies on character offsets and entity labels. There is potential for further customizing spaCy’s processing pipelines by adding more components such as tagger and parser^7^, but no established approaches in this regard have been made available partly because spaCy’s model architecture is different from those of other popular NER tools. We also observed that some entity classes, such as Gene and Protein, have zero values for precisions, recalls, and *F*_1_ scores, which likely translate to no correct classifications made for those entities. The zero values occur partly due to the relatively smaller number of tokens for those entity classes in the training set, and as a result, the trained NER model generalized poorly on the minority class entities in the test subset.

Due to spaCy’s computational demands, we did not conduct tenfold cross-validation. NERsuite provides a well-integrated pipelined system where training a new model consists of a few lines of code. In addition, NERsuite has a demonstrated record^5^ on two biomedical tasks, the BioCreative2 gene mention recognition task and the NLPBA 2004 NER task. Therefore, one could argue that it offers an advantage over spaCy for NLP tasks in specialized domains such as biomedicine.

We further applied another package, called scispaCy^8^, that contains spaCy models for processing biomedical, scientific, or clinical text. SciSpaCy acts as an extension to spaCy and provides a set of practical tools for text processing in the biomedical domain^8^. In particular, scispaCy includes a set of spaCy NER models trained on popular biomedical corpora, which covers entity types such as chemicals, diseases, cell types, proteins, and genes. As an extension to spaCy, it also has the flexibility for users to train a custom NER model from scratch or update the existing NER models with users’ own training data. Since our NER ontology adopts a more diverse and detailed annotation methodology for named entity types, it will be challenging to update scispaCy’s pretrained named entity recognizer with our annotated corpora.

Note that NERsuite was implemented in C++ and consists of three modularized programs: a tokenizer, a tagger, and a named entity recognizer^9,10^. The algorithm used behind the named entity recognizer is conditional random fields (CRF), which is often applied in tasks such as NER, part-of-speech tagging, and gene prediction. The NER model in spaCy features a sophisticated word embedding strategy using subword features and “Bloom” embeddings, a deep convolutional neural network with residual connections, and a novel transition-based approach to named entity parsing^11–13^. The Spark NLP library is inspired by a former state-of-the-art model for NER, which adopts a novel neural network architecture that automatically detects word- and character-level features using a hybrid bidirectional LSTM and CNN architecture, eliminating the need for most feature engineering^14^.

Next, we built a NER model with BERT in the Spark NLP library, which is inspired by a former state-of-the-art model for NER: Chiu & Nicols, NER with Bidirectional LSTM-CNN. The paper presents a novel neural network architecture that automatically detects word- and character-level features using a hybrid bidirectional LSTM and CNN architecture, eliminating the need for most feature engineering. The overall automated NER performance is low, with 28.2% precision, 8.4% recall, and a 12.9% *F*1 score. The best performance class, GeneOrProtein, had results of 32.3% precision, 27.2% recall, and a 29.5% *F*1 score. We observed 8 out of 13 entity classes have zero values for precision, recalls, and *F*1 scores. The zero values occur partly due to the relatively smaller number of tokens for those entity classes in the training set, and as a result, the trained NER model generalized poorly on the minority class entities in the test subset. Another reason for the large proportion of zeros in our results might be that Spark NLP requires a stricter input data format, that is, conll 2003. Therefore, further data normalization and cleaning can potentially improve upon our current results.

Finally, we compared our work against a nested NER neural learning model as proposed by Ju et al. (2018)^15^. Similar to our previous experiments, we divide the corpus into training and test data, where a full description of the results can be found in Supplementary Tables 1–3. We chose to use the nested NER for comparison, because of its ability to capture fine-grained semantic information in text by stacking NER layers. This enables the learning model to extract entities in an inside-out way using all the encoded information available^15^. Overall, the learning model achieves a 53.527% precision, 54.29% recall, and 53.906% *F*1 score, improving upon previous results using the NERsuite by 10%. The best performing class is Gene or Protein achieving 65.64% precision, 75.992% recall, and 70.439% *F*1 score.

### Word embedding experiments

Semantic associations, automatically extracted from text using neural network embedding operations, can function as a kind of “digital double” of real-world phenomena embedded in the text, facilitating inferences that were previously imagined only possible from the original experimental data. For example, word embeddings built from chemical and materials science texts predict much of the subsequent decades’ material discoveries^8^, just as the corpus of molecules can recover the periodic table^9^, and texts are able to recover the subtle, psychological, and sociological biases of cultures that produced them^16,17^. We used word embedding models to evaluate the biomedical veracity of NERO and its text annotation. Embedding models like Google’s *word2vec*^18,19^ initially received substantial attention based on their capacity to solve analogy problems and automatically capture deep semantic relationships among concepts. Building on these capacities^16,20,21^, we proposed a general method for constructing meaningful dimensions by taking the arithmetic mean of word vectors representing antonyms along a dimension and using them to diagnose their meanings. This approach has been widely validated^11,21–24^, and we employed it here to construct and compare the meanings embedded in NERO and our annotated corpus with ground truth data about drugs and diseases. In order to evaluate word embeddings based on NERO, we identified two disease properties —(1) severity and (2) gender specificity—and likewise two therapeutic drug properties —(1) toxicity and (2) expense—not directly present in the text, but highly relevant to diagnosis and treatment, and on which text-independent ground truth data exists.

**Fig. 3:**
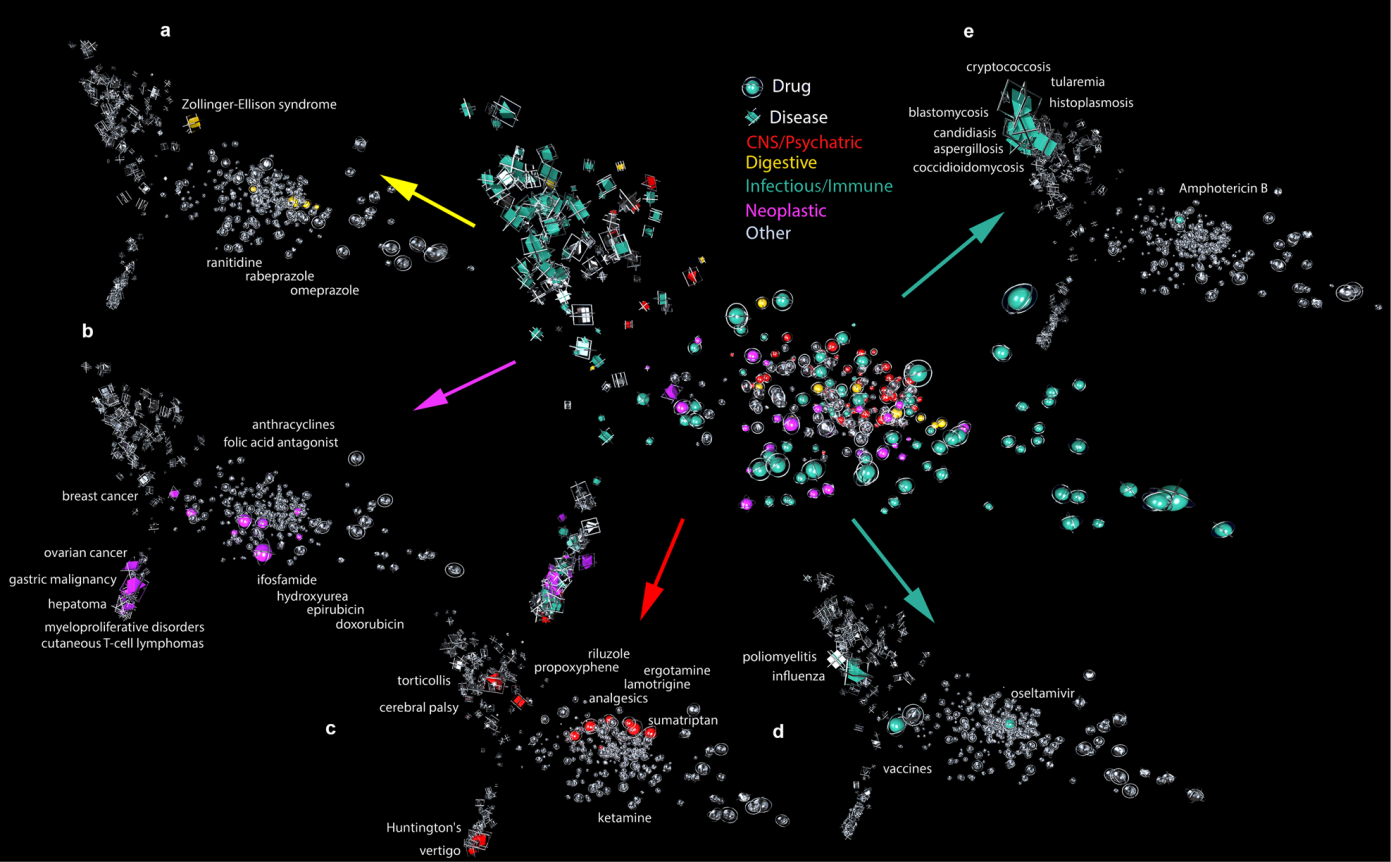
Projection of text embedding into three-dimensional space. Properties of diseases and drugs are visible in the first three principal components of our multi-dimensional text embedding. The figure shows a projection of text embedding into three-dimensional space, with named entities corresponding to diseases and drugs shown with prisms and spheres, respectively. The figure represents several projections of the same embedding, preserving spatial layout and projection, with distinct elements of the embedding indicated by shape color. The central image shows all disease systems and their corresponding medications together. More specifically, the additional projections show: **a** Zollinger–Ellison syndrome and associated medications; **b** cancers and associated therapies; **c** central nervous system diseases and corresponding medications; **d**, **e** Viral and bacterial infectious diseases, respectively, together with corresponding antiviral and antibiotic agents, and **f** 3-dimensional projection of embedding drug- and disease-related named entities corresponding to CNS/Psychiatric- (red), digestive- (yellow), infectious/immune- (green), neoplastic- (cyan), and other diseases (grey). Another view of the same dataset is presented in Fig. 4.

We embedded named entities associated with diseases and drugs into a high-dimensional space in which every NERO term was assigned a 300-dimensional vector, (see Fig. 3 for a three-dimensional projection of this embedding), along with a selection of diseases and medications used to treat them. For all embedding experiments, we used a word2vec^18,19^ implementation provided by the package gensim^25^. The corpus that we used included (1) English Wikipedia download; (2) A collection of articles from 15 Elsevier journals licensed to the University of Chicago; and (3) A collection of Reuters newswire articles purchased from the provider. The corpus was represented as a shuffled set of sentences stored in an Oracle Berkeley DB, allowing dynamic reordering of sentences, followed by feeding one sentence at a time into a word2vec bag of words model. For incorporating disease-specific vocabulary we used the dis2vec biomedical wrapper for gensim^12^. The parameters used for analysis included an embedding dimension (300, but we also tried 100 and 500 with results essentially unchanged), a sentence window size of ten words, and training run off 20 epochs.

We then compared drug and disease projections into the embedding dimensions for severity, gender, toxicity, and expense with ground truth about these qualities. We constructed the severe-mild axis with the following contrasting term pairs: (harmful, beneficial), (serious, benign), (life-altering, common), (disruptive, undisruptive), (dying, recovering), (dangerous, safe), (threatening, low-priority), (high mortality, harmless), (costly, cheap), (hospitalized, self-administered), (hospital, work), (debt, savings), (low quality of life, undisruptive), and (hazard, routine). Then we compared disease projection in this dimension with World Health Organization data on the burden of living with each of those diseases (DALYs^13^) and found a correlation of 0.329 (*p* = 0.0614*, n* = 33). We then constructed a gender dimension with similarly contrasting pairs: (male, female), (prostate, ovary), (penile, uterine), (penis, uterus), (man, woman), (men, women), (masculine, feminine), (he, she), (him, her), (his, hers), (boy, girl), and (boys, girls). We compared the disease projection in this gender dimension with the prevalence of those diseases for men and women from a substantial sample of doctor–patient insurance records capturing ~47% of all of US doctor–patient visits between 2003 and 2011 and found a correlation of 0.436 (*p* = 1.46 × 10^−13^, *n* = 261).

We tested the robustness of our 300-dimensional embedding by comparing it with 100-dimensional and 500-dimensional embeddings obtained using the same corpus and comparing distances between the same pair of named entities (disease or drug) in embeddings of different dimensionality. The results appear to be very stable with respect to dimensionality of the embedding: distances between named entities were highly correlated at *ρ* = 0.89 for 100- and 300-dimensional embeddings comparison, and at *ρ* = 0.95 for 500- to 300-dimensional embedding comparison (see Fig. 4 in the Supplementary Data).

**Fig. 4:**
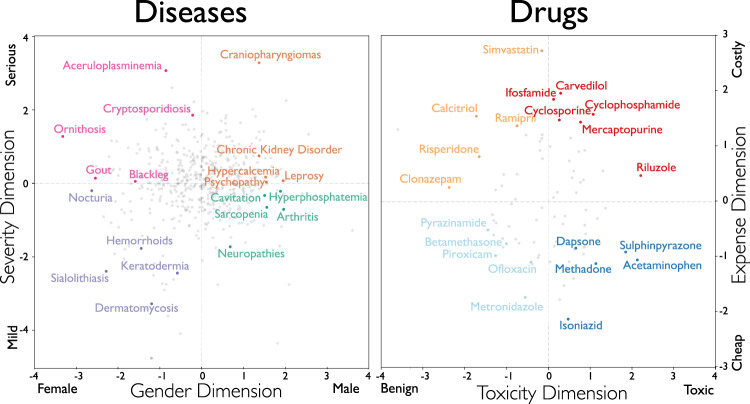
Two-dimensional projections of diseases and medications. Left We projected diseases into two dimensions: female-male (X-axis) and severe-mild (Y-axis). We defined the “male–female” axis using the following pairs of terms: (“male,” “female”), (“prostate,” “ovary”), (“penile,” “uterine”), (“penis,’’ “uterus”), (“man,” “woman”), (“men,” “women”), (“masculine,” “feminine”), (“he,“ “she”), (“him,” “her”), (“his,” “hers”), (“boy,” “girl”), and (“boys,” “girls”). We defined the severe-mild axis with the following term pairs: (“harmful,” “beneficial”), (“serious,” “benign”), (“life-altering,” “common“), (“disruptive,” “undisruptive”), (“dying,’’ “recovering”), (“dangerous,” “safe”), (“threatening,” “low-priority”), (“high mortality,” “harmless”), (“costly,” “cheap”), (“hospitalized,” “self-administered”), (“hospital,” “work”), (“debt,” “savings”), (“low quality of life,” “undisruptive”), and (“hazard,” “routine'). Right We projected medications into “benign-toxic” (X-axis) and “cheap-costly” (Y-axis). For the “benign-toxic” axis, we used the following pairs of antonym words: (“harmful,” “beneficial”), (“toxic,” “nontoxic”), and (“noxious,” “benign”). We defined the “expensive–inexpensive” dimension using the following pairs of terms: (“expensive,” “inexpensive“), (“costly,” “cheap”), (“brand,” “generic”), and (“patented,” “off-patent”).

Next, we projected medication-related NERs onto a toxicity axis composed from the following antonym pairs: (harmful, beneficial), (toxic, nontoxic), and (noxious, benign) and an expense dimension anchored by: (expensive, inexpensive), (costly, cheap), (brand, generic), and (patented, off-patent). The embedding-derived drug projections onto the toxic-harmless dimension correlates at 0.32 (*p* = 1.1 × 10^−4^) with the corresponding drug-specific median lethal dose (dose required to kill 50% of model animals as documented in the LD50 database^26^). Finally, the correlation of drug projections into an expense dimension and the actual price of each drug as listed in the IBM MarketScan database^27^ was 0.42 (*p* = 1.5 × 10^−15^) (see Fig. 4). When a disease projects low in the *male–female* dimension, it is much more likely to afflict women than men, such as ornithosis and related infectious diseases. When a disease projects high in the *serious–benign* dimension like leprosy, it is likely to incur substantial suffering. Medications projecting with negative (toxic) values in the toxic–nontoxic dimension, as the name suggests, tend to be associated with more severe side effects. For example, the drug Riluzole, a treatment for amyotrophic lateral sclerosis, has side effects ranging from unusual bleeding to nausea and vomiting. Drug projections high in the *expensive–inexpensive* dimension forecast a stiff medical bill, as in the case of Simvastatin, which is used to reduce the risk of heart attack and stroke, and which, before it went off-patent, used to cost hundreds of dollars per bottle. The robustness of these results suggests that scientific corpora can be used for the automated generation of robust hypotheses meriting follow-up empirical study.

## Discussion

This study’s main limitation is that, even though our NERO ontology aimed to cover all entities contained in the biomedical research literature, we did not cover all levels of granularity in classifying entities. Moreover, while the major concepts are well-annotated, several concept types were not well-represented because of the heavy-tail distribution of ontological class frequencies. In addition, we note that satisfactory results of NER rely heavily on a large quantity of hand-annotated data, which is often costly in terms of time and resources spent. Therefore, the adoption of semi-supervised learning methods, which incorporates unlabeled data to improve learning accuracy, could reduce the need for manual annotation^28^.

While there is a popular belief that pretraining on general-domain text can be helpful for developing domain-specific language models, a recent study has shown that for specialized domains, such as biomedicine, pretraining on in-domain text from scratch offers noticeable improvements in model accuracy compared to continual pretraining of general-domain language models^29^. Therefore, we trained our annotated corpus from scratch in our machine learning experiments^30^.

The resources offered in our study can be applied to a wide range of scientific problems. First, the proposed NERO ontology can facilitate more robust and accurate large-scale text mining of biomedical literature. As discussed above, NERO is the first knowledge graph in this field, accounting for context-relevant levels of ambiguity. Graph neural networks^31^ can leverage such prior knowledge from human experts for learning embeddings of biomedical entities, which is likely to preserve both semantic meaning in the original literature and domain knowledge from human experts. Second, researchers can combine the curated corpus from this study with self-supervised learning^32^. Such a learning scenario can utilize the unlabeled data in a supervised way by predicting part of the sentence using the rest of the sentence. The annotated corpus from this study can be used to fine-tune language models, orienting them for critical biomedical tasks.

## Methods

Snippets of methods are integrated with results.

## Supplementary information

Supplementary Information

## Data Availability

The datasets generated during and/or analyzed during the current study are available in the Github repository at https://github.com/arzhetsky/Chicago_corpus. NERO in OWL format is available at: https://bioportal.bioontology.org/ontologies/NERO.

## Code availability

We also deployed a package called NERO-nlp for researchers interested in diving deeper into our annotated corpus; the installation guides and scripts are available online at https://pypi.org/project/NERO-nlp and https://github.com/Bohdan-Khomtchouk/NERO-nlp, respectively.
